# LncRNA NEAT1 Regulates Cell Viability and Invasion in Esophageal Squamous Cell Carcinoma through the miR-129/CTBP2 Axis

**DOI:** 10.1155/2017/5314649

**Published:** 2017-09-25

**Authors:** Yong Li, Dong Chen, Xiang Gao, Xiaohui Li, Gongning Shi

**Affiliations:** Department of Cardiothoracic Surgery, Huaihe Hospital of Henan University, Kaifeng 475000, China

## Abstract

**Background:**

Long noncoding RNA nuclear paraspeckle assembly transcript 1 (NEAT1) was reported to be aberrantly upregulated and promote esophageal squamous cell carcinoma (ESCC) cell progression. Nevertheless, the molecular mechanism of NEAT1 involved in the competing endogenous RNA (ceRNA) regulatory network in ESCC progression remains poorly defined.

**Methods:**

The expressions of NEAT1, miR-129, and C-terminal-binding protein 2 (CTBP2) in ESCC cells were examined by qRT-PCR. The effects of NEAT1 knockdown and miR-129 overexpression, or along with CTBP2 upregulation, on ESCC cell viability and invasion were explored by CCK-8 and transwell invasion assays, respectively. Luciferase reporter assay in combination with RIP was performed to confirm the interaction between NEAT1, miR-129, and CTBP2.

**Results:**

NEAT1 and CTBP2 were upregulated and miR-129 was downregulated in ESCC cells. Either NEAT1 knockdown or miR-129 overexpression suppressed ESCC cell viability and invasion. Moreover, NEAT1 functioned as an endogenous sponge to downregulate miR-129 by competitively binding to miR-129, thereby leading to the derepression of CTBP2, a target of miR-129. CTBP2 restoration overturned cell viability and invasion suppression mediated by NEAT1 knockdown or miR-129 overexpression.

**Conclusion:**

LncRNA NEAT1 regulated ESCC cell viability and invasion via the miR-129/CTBP2 axis, contributing to the better understanding of the molecular mechanism of ESCC pathogenesis and progression.

## 1. Introduction

Esophageal squamous cell carcinoma (ESCC) is the predominant histological type of esophageal cancer (EC) and is considered one of the most common and leading aggressive malignancies all around the world, with an unexpectedly high fatality rate [1]. Clinically, despite remarkable advances in surgical techniques and treatment, the overall prognosis of patients with ESCC is still unfavorable, with a five-year survival of less than 10%, and the median survival of late-stage patients is less than 1 year [2, 3]. Due to no specific symptoms in an early stage, the majority of patients with ESCC are often diagnosed at an advanced stage with extensive local invasion and regional lymph node metastasis [4, 5]. Thus, identification of new accurate molecular markers for early diagnosis and a better understanding of the crucial molecular mechanisms of esophageal carcinogenesis are highly desirable for improving the prognosis and survival of ESCC patients.

Long noncoding RNAs (lncRNAs) are identified as a new class of evolutionarily conserved RNA molecules longer than 200 nucleotides without or with limited protein-coding capacity [6]. Mounting evidence suggests that lncRNAs play a wide range of functional roles in a variety of biological processes, such as development, growth, invasion, metastasis, and tumorigenesis [7, 8]. Additionally, dysregulation of lncRNAs has been linked to the initiation and development of numerous tumors, including ESCC [9–11]. LncRNA HOTTIP was reported to be upregulated in ESCC, and knockdown of HOTTIP significantly inhibited ESCC cell proliferation and invasion [12]. LncRNA CASC9 was found to be markedly elevated in ESCC tissues, and downregulation of CASC9 suppressed ESCC cell migration and invasion [13]. LncRNA UCA1 was demonstrated to block ESCC growth by decreasing cell proliferation, migration, invasion, and cell cycle progression through regulating the Wnt signaling pathway [14]. Although considerable attentions and great efforts have been put into the ways by which lncRNAs affect cancer progression, their abnormal expression and functional roles in ESCC development are far from being fully elucidated. Long noncoding RNA nuclear paraspeckle assembly transcript 1 (NEAT1), the core structural component of the nuclear body paraspeckle, was initially transcribed from the familial tumor syndrome multiple endocrine neoplasia locus [15]. It is well documented that NEAT1 is implicated in the tissue development of mouse mammary gland [16] and corpus luteum [17]. Moreover, NEAT1 displays carcinogenicity in multiple types of cancers, such as breast cancer [18] and glioma [19]. In a recent publication, NEAT1 was reported to be aberrantly upregulated and promote ESCC progression by stimulating cell proliferation and enhancing cells' ability of forming foci, migration, and invasion [20].

Over the past several years, increasing studies are focusing on the lncRNA-miRNA-mRNA regulatory mechanism that lncRNAs may serve as competing endogenous RNAs (ceRNAs), namely, miRNA sponges or antagomirs, to negatively regulate miRNAs, eventually resulting in the derepression of miRNA targets [21]. It was previously reported that NEAT1 epigenetically silenced miR-129-5p expression by promoting DNA methylation of the CpG island in the miR-129 gene in breast tumorigenesis, thus resulting in an upregulation of WNT4 expression, a target of miR-129-5p [22]. Moreover, overexpression of NEAT1 inhibited the expression of miR-129-5p in hepatocellular carcinoma by regulating its targets, namely, valosin-containing protein (VCP) and inhibitor of kappa B (I*κ*B) [23]. Therefore, we hypothesized that NEAT1 and miR-129 were involved in the ceRNA regulatory network in ESCC progression.

C-terminal-binding protein 2 (CTBP2) is a member of CTBP family protein located at human chromosome 10 that is conserved among both vertebrates and invertebrates [24]. CTBP2 is well known to function as a transcriptional corepressor and modulator of several essential tumorigenic processes, including growth, proliferation, and invasion, in a variety of cancer cells [25]. Accumulating evidence indicated that CTBP2 was aberrantly upregulated in several types of malignancy and promoted tumorigenesis and progression [26]. Moreover, CTBP2 was demonstrated to be highly expressed in ESCC tumor tissues and contribute to the progression of ESCC through negatively regulating p16 (INK4A) [27].

According to bioinformatic analysis data, CTBP2 was predicted to be one of the potential targets of miR-129. Therefore, we aimed to study the relationship between NEAT1, CTBP2, and miR-129 and investigate whether the NEAT1/miR-129/CTBP2 axis was implicated in the progression of ESCC. In the present study, we evaluated the expression and function of NEAT1, miR-129, and CTBP2 in ESCC. Furthermore, mechanistic analysis demonstrated that NEAT1 served as a ceRNA by sponging miR-129 to upregulate CTBP2, thus promoting ESCC cell proliferation and invasion. Our study first provided the evidence for the cross-talk between NEAT1, miR-129, and CTBP2, contributing to the diagnosis and therapy for ESCC.

## 2. Materials and Methods

### 2.1. Cell Lines and Culture Conditions

A human esophageal epithelial cell line (HET-1A) and human ESCC cell lines (EC109 and EC9706) were all provided by the American Type Culture Collection (ATCC, Manassas, VA, USA). All cells were maintained in RPMI 1640 medium (Invitrogen, Carlsbad, CA, USA) containing 10% fetal bovine serum (FBS; Gibco, Grand Island, NY, USA), 100 U/ml penicillin, and 100 *μ*g/ml streptomycin (Hyclone, Logan, UT, USA) at 37°C with humidified atmospheres of 5% CO_2_.

### 2.2. Cell Transfection

A CTBP2 overexpression vector was constructed by inserting full-length CTBP2 into pcDNA3.1 (pcDNA, Invitrogen). siRNA against NEAT1 (si-NEAT1), miR-129 mimics (miR-129), miR-129 inhibitor (anti-miR-129), and the scramble negative controls (si-control, miR-control, and anti-miR-control) were designed and synthesized from RiboBio (Guangzhou, China). EC109 and EC9706 cells were seeded in 96-well plates at a density of 5 × 10^4^ cells/well 24 h before transfection. Lipofectamine 2000 reagent (Invitrogen) was used to transfect oligonucleotides or plasmids into cells.

### 2.3. RNA Extraction and Quantitative Real-Time PCR (qRT-PCR)

Total RNA was extracted from cultured cells using TRIzol reagent (Invitrogen) according to the manufacturer's protocol. A total of 5 *μ*g RNA was reversely transcribed into cDNA with a High-Capacity cDNA Reverse Transcription Kit (Applied Biosystems, Foster City, CA, USA). A TaqMan miRNA Reverse Transcription Kit (Applied Biosystems) was applied to synthesize cDNA of miRNAs. The expression levels of NEAT1, miR-129, and CTBP2 mRNA were quantified using the SYBR® Premix Ex Taq™ (Takara, Dalian, China) on the Roche LightCycler 480 Real-Time PCR System (Applied Biosystems). U6 small RNA was used as the internal reference for miR-129, and GAPDH was chosen as the endogenous control for NEAT1 and CTBP2 mRNA. The relative gene expression was calculated using the 2^−ΔΔCt^ method. The primers used in this study were synthesized by Invitrogen and presented as follows: NEAT1, 5′-ATGCCACAACGCAGATTGAT-3′ (forward) and 5′-CGAGAAACGCACAAGAAGG-3′ (reverse); miR-129, 5′-GCGACTGA CGTCTTTTTGCGGTCTGG-3′ (forward) and 5′-CAGA ACAGTGTCGTGACAGTGACGAT-3′ (reverse); CTBP2, 5′-ATCCACGAGAAGGTTCTAAACGA-3′ (forward) and 5′-CCGCACGATCACTCTCAGG-3′ (reverse); U6, 5′-GCTTCGGCAGCACATATACTAAAAT-3′ (forward) and 5′-CGCTTCACGAATTTGCGTGTCAT-3′ (reverse); and GAPDH, 5′-TATGATGATATCAAGAGGGTAGT-3′ (forward) and 5′-TGTATCCAAACTCATTGTCATAC-3′ (reverse).

### 2.4. Western Blot Analysis

Total protein from cells was isolated by using protein extraction reagent RIPA buffer (50 mM Tris-HCl, 150 mM NaCl, 2 mM EDTA, 1% NP-40, and 0.1% SDS) on ice and quantified by the BCA Protein Assay Kit (Beyotime, China). The protein samples were subjected to 10% sodium dodecyl sulfate-polyacrylamide gel electrophoresis (SDS-PAGE) and then transferred to a nitrocellulose membrane (Millipore, Bedford, MA, USA). Following blocking with 5% skim milk at room temperature for 1 h, the immunoblots were incubated with monoclonal antibody against CTBP2 (1 : 500 dilution, Santa Cruz Biotechnology, Santa Cruz, CA, USA) and *β*-actin (1 : 1000 dilution, Sigma, St. Louis, MO, USA) as the internal control. Subsequently, the membranes were incubated with the horseradish peroxidase- (HRP-) conjugated secondary antibody anti-rabbit IgG (1 : 1000 dilution, Santa Cruz Biotechnology) for 1 h. The protein signals were determined using the ECL detection kit (Pierce Biotechnology, Rockford, IL, USA).

### 2.5. Cell Counting Kit-8 (CCK-8) Assay

Cell viability was measured by CCK-8 (Beyotime Institute of Biotechnology, Jiangsu, China). In brief, the transfected EC109 and EC9706 cells were seeded into 96-well plates at a density of 2000 cells/well and cultured for 48 h. Subsequently, 10 *μ*l CCK-8 solution was added to each well of the plate and incubated at 37°C for 2 h. The optical density at 450 nm, which is indicative of a positive correlation with cell viability, was measured using a microplate reader (Elx800; BioTek Inc., North Brunswick, NJ, USA).

### 2.6. RNA Immunoprecipitation (RIP)

To verify the relationship between NEAT1 and miR-129, RIP was conducted using the Magna RIP™ RNA-Binding Protein Immunoprecipitation Kit (Millipore). Briefly, EC109 and EC9706 cells at 80% confluency were harvested and lysed in complete RIP lysis buffer. Afterwards, the whole cell extract (100 *μ*l) was coimmunoprecipitated with RIP buffer containing magnetic beads conjugated with anti-Argonaute2 (Ago2) antibody (Millipore) or normal mouse IgG (Millipore) as a negative control. Then, samples were digested with proteinase K, and then, coprecipitated RNA was isolated and subjected to qRT-PCR analysis of NEAT1 and miR-129.

### 2.7. Luciferase Reporter Assay

The wild-type NEAT1 (NEAT1-WT), mutant NEAT1 (NEAT1-MUT), wild-type CTBP2-3′UTR (CTBP2-WT), and mutant CTBP2-3′UTR (CTBP2- MUT) were synthesized and cloned into pMIR-GLO™ Luciferase vectors (Promega, Madison, WI, USA). Luciferase reporter constructs containing the wild-type or mutated miR-129 (pMIR-miR-129-WT and pMIR-miR-129-MUT) were purchased from GeneChem (Shanghai, China). For the luciferase reporter assay, EC109 and EC9706 cells were cotransfected with constructed luciferase reporter vectors containing NEAT1 (WT or MUT) or CTBP2-3′UTR (WT or MUT) and miR-129 or miR-control. To investigate the effects of NEAT1 on wild-type or mutated miR-129, EC109 and EC9706 cells were cotransfected with miR-129-WT or miR-129-MUT reporter and pcDNA-NEAT1 or pcDNA. To determine the relationship between NEAT1, miR-129, and CTBP2, EC109 and EC9706 cells were transfected with CTBP2-WT, CTBP2-WT + miR-129, or along with pcDNA-NEAT1 or pcDNA. Lipofectamine 2000 reagent (Invitrogen) was utilized for all cell transfections. At 48 h posttransfection, cells were collected and assayed with the Dual-Luciferase Reporter Assay System (Promega). Firefly luciferase activity was normalized to that of Renilla luciferase.

### 2.8. Cell Invasion Assay

The 24-well transwell chambers (Corning Incorporated, Corning, NY, USA) coated with 8 *μ*M pore-size Matrigel (BD Biosciences, Franklin Lakes, NJ, USA) were used to assess the invasiveness of ESCC cells. The transfected EC109 and EC9706 cells (1 × 10^4^ cells) were resuspended in 150 *μ*l serum-free RPMI-1640 medium and seeded onto the top of the invasion chambers. The lower chambers were filled up with 600 *μ*l RPMI-1640 containing 10% fetal bovine serum as a chemoattractant. After 24 h incubation at 37°C, noninvasive cells inside the upper chamber were scraped off with cottons swabs and invading cells on the lower membrane surface were fixed in 4% paraformaldehyde for 15 min and then stained with 0.3% crystal violet for 15 min. Cells were photographed and counted in ten fields at 100x magnification using a microscope (Nikon, Tokyo, Japan).

### 2.9. Statistical Analysis

Data were presented as mean ± standard deviation (SD) from at least three experiments. All statistical analyses were performed using GraphPad Prism V5.0 software (GraphPad Software Inc., La Jolla, CA, USA). The significant differences were analyzed using Student's *t*-test or one-way ANOVA. *P* values less than 0.05 were considered to indicate a statistically significant difference.

## 3. Results

### 3.1. NEAT1 and CTBP2 Were Upregulated and miR-129 Was Downregulated in ESCC Cells

**Figure 1:**
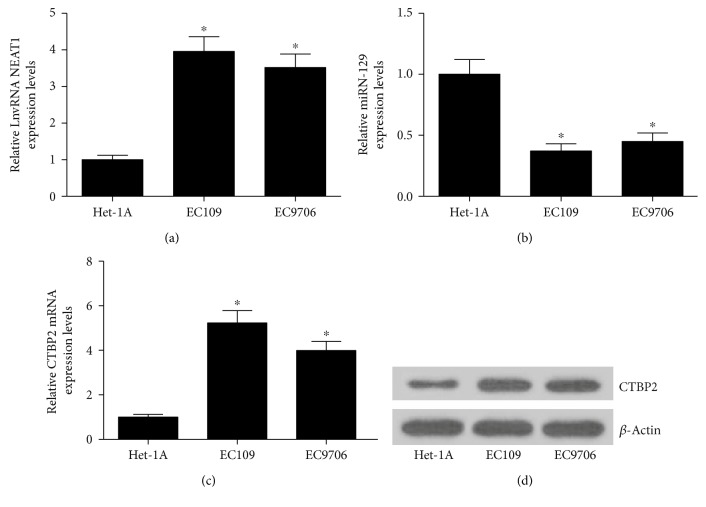
Expressions of NEAT1, miR-129, and CTBP2 in ESCC cells. qRT-PCR analysis of expression levels of NEAT1 (a), miR-129 (b), and CTBP2 mRNA (c) in a human esophageal epithelial cell line (HET-1A) and ESCC cell lines (EC109 and EC9706). (d) The protein level of CTBP2 in HET-1A, EC109, and EC9706 was detected by Western blot. ^∗^*P* < 0.05.

We first examined the expressions of NEAT1, miR-129, and CTBP2 in ESCC cell lines (EC109 and EC9706). As compared with the human esophageal epithelial cell line HET-1A, the expressions of NEAT1 (Figure 1(a)) and CTBP2 at mRNA (Figure 1(c)) and protein (Figure 1(d)) levels were all significantly elevated in EC109 and EC9706 cells. Additionally, it is evident that miR-129 was markedly downregulated in EC109 and EC9706 cells versus HET-1A cells (Figure 1(b)).

### 3.2. NEAT1 Knockdown or miR-129 Overexpression Suppressed ESCC Cell Viability and Invasion

**Figure 2:**
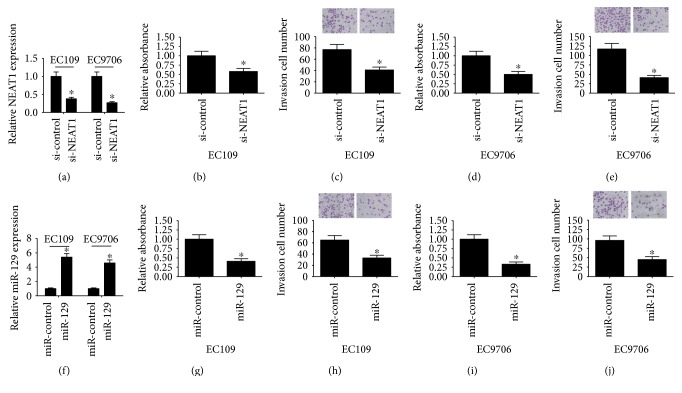
Effects of NEAT1 knockdown and miR-129 overexpression on ESCC cell viability and invasion. (a) qRT-PCR analysis of NEAT1 expression in si-NEAT1-transfected EC109 and EC9706 cells. (f) qRT-PCR analysis of miR-129 expression in EC109 and EC9706 cells introduced with miR-129 mimic. CCK-8 assay was applied to determine cell viability in EC109 (b, g) and EC9706 (d, i) cells transfected with si-NEAT1, si-control, miR-129, or miR-control. Transwell invasion assay was carried out to assess cell invasiveness in EC109 (c, h) and EC9706 (e, j) cells transfected with si-NEAT1, si-control, miR-129, or miR-control. ^∗^*P* < 0.05.

To explore the biological functions of NEAT1 and miR-129 in ESCC progression, we performed NEAT1 knockdown or miR-129 overexpression experiments in EC109 and EC9706 cells. qRT-PCR analysis demonstrated that NEAT1 expression was downregulated in si-NEAT1-transfected ESCC cells (Figure 2(a)), while miR-129 expression was upregulated following introduction of miR-129 mimic in both EC109 and EC9706 cells (Figure 2(f)). CCK-8 results showed that cell viability was effectively inhibited in EC109 and EC9706 cells transfected with si-NEAT1 (Figures 2(b) and 2(d)) or miR-129 mimic (Figures 2(g) and 2(i)) compared with si-control or miR-control groups. As demonstrated by cell invasion assay, NEAT1 knockdown (Figures 2(c) and 2(e)) or forced expression of miR-129 (Figures 2(h) and 2(j)) led to a significant reduction in cell invasiveness in EC109 and EC9706 cells compared to respective control groups. Based on these results, we proposed that abnormal expression of NEAT1 or miR-129 was associated with ESCC progression.

### 3.3. NEAT1 Functioned as an Endogenous Sponge to Downregulate miR-129 by Competitively Binding to miR-129

**Figure 3:**
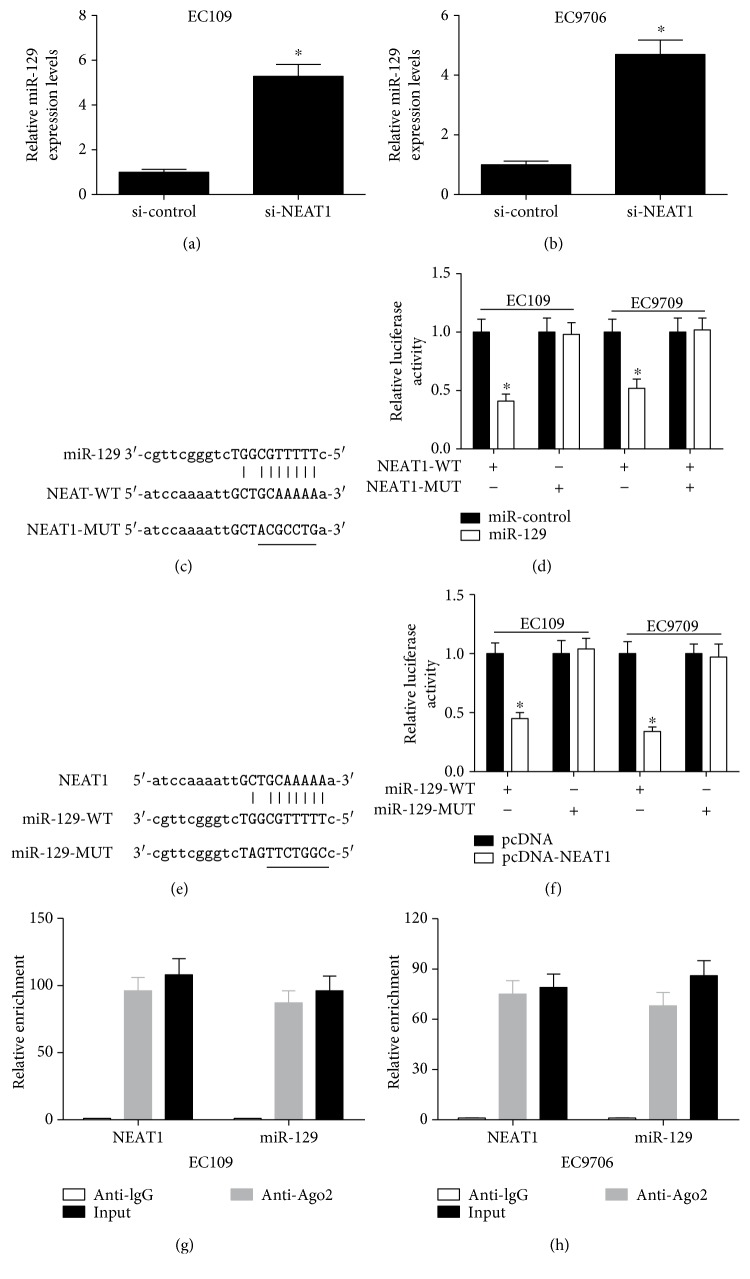
NEAT1 functioned as an endogenous sponge to downregulate miR-129 by competitively binding to miR-129. qRT-PCR was performed to detect the expression of miR-129 in EC109 (a) and EC9706 (b) cells transfected with si-NEAT1 or si-control. (c, e) The predicted binding sites between miR-129 on NEAT1, as well as the mutants of NEAT1 and miR-129. (d) The relative luciferase activity in EC109 and EC9706 cells cotransfected with luciferase reporter vectors containing NEAT1-WT or NEAT1-MUT and miR-control or miR-129. (f) The relative luciferase activity in EC109 and EC9706 cells cotransfected with miR-129-WT or miR-129-MUT reporter and pcDNA or pcDNA-NEAT1. RIP assay was conducted in EC109 (g) and EC9706 (h) cell extracts to examine miR-129 endogenously associated with NEAT1. ^∗^*P* < 0.05.

The ceRNA hypothesis points that lncRNA functions as a molecular sponge of miRNA to liberate mRNA transcript targeted by miRNA, thereby affecting tumorigenesis and cancer progression [28]. To determine whether NEAT1 had a similar mechanism in ESCC, qRT-PCR was first used to investigate the effect of NEAT1 knockdown on miR-129 expression in ESCC cells. As shown in Figures 3(a) and 3(b), miR-129 expression was distinctly increased after transfection of si-NEAT1 in EC109 and EC9706 cells. Thus, we speculated that NEAT1 acted as a molecular sponge of miR-129 in ESCC. Accordingly, bioinformatic analysis of potential miRNAs binding to NEAT1 was performed using the online software program starBase v2.0 [29]. As expected, NEAT1 was predicted to contain a motif with sequence complementary to miR-129 (Figures 3(c) and 3(e)). To further confirm the direct interaction between NEAT1 and miR-129, we constructed luciferase reporter vectors containing wild-type (WT) or mutated (MUT) NEAT1 (Figure 3(c)) or miR-129 (Figure 3(e)) and performed luciferase reporter assay. As illustrated in Figure 3(d), ectopic expression of miR-129 led to a marked reduction in luciferase activity of NEAT1-WT but had no evident inhibitory effect on NEAT1-MUT in EC109 and EC9706 cells. Also, NEAT1 overexpression significantly inhibited the luciferase activity of pMIR-miR-129-WT reporter but not that of pMIR-miR-129-MUT reporter in EC109 and EC9706 cells (Figure 3(f)). These results demonstrated that NEAT1 could mutually interact with miR-129 in ESCC cells. A previous study demonstrated that lncRNA could also negatively regulate miRNA expression by associating with Ago2-containing RNA-induced silencing complex (RISC) [30]. To further validate the mutual effect of NEAT1 and miR-129 at endogenous levels, we performed RIP assay to pull down endogenous miRNAs associated with NEAT1 in EC109 and EC9706 cells using antibody against Ago 2. Consistent with bioinformatic analysis and luciferase assay, we found that NEAT1 and miR-129 were both specifically enriched in Ago2 pellets of EC109 (Figure 3(g)) and EC9706 (Figure 3(h)) cell extracts relative to the IgG control group. Together, these results implied that NEAT1 could directly bind to miR-129 and serve as a ceRNA.

### 3.4. NEAT1 Derepressed CTBP2 by Inhibiting miR-129 Expression

**Figure 4:**
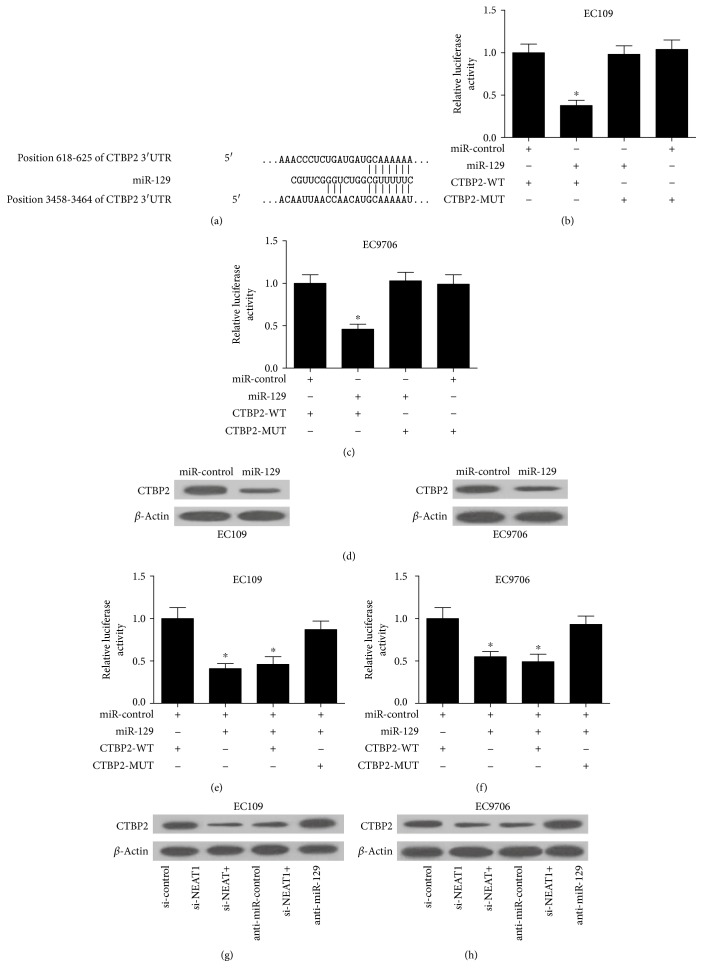
NEAT1 regulated CTBP2 expression by competitively binding to miR-129. (a) Putative binding regions between CTBP2 and miR-129. The relative luciferase activity of EC109 (b) and EC9706 (c) cells cotransfected with pMIR luciferase reporter vectors containing wild-type or mutated CTBP2 3′UTR and miR-129 or miR-control. (d) Western blot analysis of CTBP2 expression in EC109 and EC9706 cells transfected with miR-129 or miR-control. The relative luciferase activity of EC109 (e) and EC9706 (f) cells cotransfected with CTBP2-WT, CTBP2-WT + miR-129, or in combination with pcDNA or pcDNA-NEAT1. The protein level of CTBP2 in EC109 (g) and EC9706 (h) cells transfected with si-NEAT1, si-control, si-NEAT1 + anti-miR-control, or si-NEAT1 + anti-miR-129. ^∗^*P* < 0.05.

To further investigate the molecular mechanism by which NEAT1 and miR-129 exerted their regulatory role in ESCC, bioinformatic-based target prediction analysis by TargetScan (http://www.targetscan.org) and miRanda (http://www.microrna.org) was performed to explore the potential targets of miR-129 in ESCC cells. As displayed in Figure 4(a), CTBP2 was predicted to contain binding sequences of miR-129. CTBP2, a member of the CTBP family, acts as a transcriptional corepressor to modulate cancer cell growth and tumorigenesis by interacting with the C-terminus of the adenoviral E1A oncoprotein [26]. Luciferase reporter assay was further used to confirm the bioinformatic prediction. The luciferase reporter vectors containing wild-type or mutated CTBP2 3′UTR were cotransfected with miR-129 or miR-control into EC109 and EC9706 cells. As shown in Figures 4(b) and 4(c), transfection of miR-129 led to a significant decrease in luciferase activity of the pMIR luciferase reporter containing WT 3′UTR of CTBP2, but not the mutant reporter. In parallel, exogenous expression of miR-129 effectively suppressed the protein level of CTBP2 in EC109 and EC9706 cells, as demonstrated by Western blot (Figure 4(d)). These data confirmed that miR-129 directly targeted CTBP2 and regulated its expression. However, cotransfection of miR-129 and pcDNA-NEAT1 recuperated the luciferase activity of the pMIR-CTBP2-WT luciferase reporter suppressed by single miR-129 transfection (Figures 4(e) and 4(f)). Furthermore, we analyzed the effect of NEAT1 silencing or miR-129 inhibitor on the expression level of CTBP2 by Western blot. The results indicated that NEAT1 knockdown remarkably reduced the protein level of CTBP2 in EC109 (Figure 4(g)) and EC9706 (Figure 4(h)) cells, while anti-miR-129 apparently abolished this effect. Taken together, these results demonstrated that NEAT1 liberated CTBP2 by competitively binding to miR-129.

### 3.5. CTBP2 Restoration Overturned the Suppression of Cell Viability and Invasion Mediated by NEAT1 Knockdown or miR-129 Overexpression

**Figure 5:**
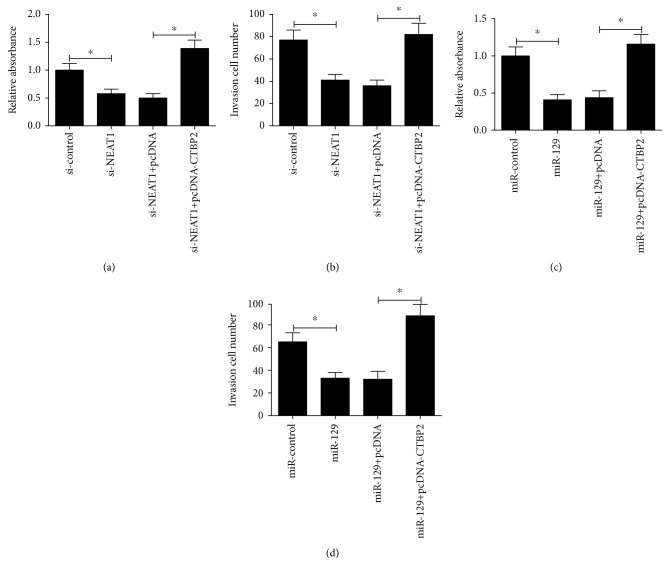
CTBP2 restoration overturned NEAT1 knockdown- or miR-129 overexpression-induced suppressive role on viability and invasion of ESCC cells. (a, b) EC109 cells were transfected with si-NEAT1, si-control, si-NEAT1 + pcDNA, or si-NEAT1 + pcDNA-CTBP2; then, CCK-8 and transwell invasion assays were performed to determine cell viability and invasiveness. (c, d) EC109 cells were transfected with miR-129, miR-control, miR-129 + pcDNA, or miR-129 + pcDNA-CTBP2; then, CCK-8 and transwell invasion assays were conducted to measure cell viability and invasiveness. ^∗^*P* < 0.05.

Based on the above findings, we infer that the NEAT1-miR-129-CTBP2 regulatory axis may be implicated in the development of ESCC. To validate this hypothesis, we further performed rescue experiments by transfecting si-NEAT1 or miR-129 mimic in combination with pcDNA-CTBP2 into EC109 cells. At indicated time points, CCK-8 and transwell invasion assays were carried out. As expected, knockdown of NEAT1 or miR-129 overexpression obviously impeded cell viability (Figures 5(a) and 5(c)) and invasiveness (Figures 5(b) and 5(d)) of EC109 cells, while CTBP2 restoration significantly abrogated these effects, suggesting that NEAT1 promoted cell viability and invasion by competitively binding to miR-129, upregulating CTBP2, and then contributing to tumorigenesis of ESCC.

## 4. Discussion

Amounting evidence shows that lncRNAs are frequently aberrantly expressed in a variety of tumors and emerging as a crucial regulator of pathological processes related to tumorigenesis, invasion, and metastasis [31, 32]. Previous reports have shed light on the biological functions of lncRNAs, as well as on the underlying molecular mechanism by which lncRNAs involve in numerous types of human cancers [33, 34]. A previous study demonstrated that NEAT1 functioned as an oncogenic lncRNA in ESCC cells [20]; however, the exercise mechanism behind NEAT1 contributing to ESCC progression has not been elucidated. Our study provided an insight into the molecular mechanism of NEAT1 in ESCC progression and identified that the dysregulation of the NEAT1/miR-129/CTBP2 axis accounted for ESCC progression.

In our study, we analyzed the biological role of NEAT1, miR-129, and CTBP2 in ESCC cells. NEAT1 was confirmed to be upregulated in ESCC cells, and NEAT1 silencing suppressed ESCC cell viability and invasion, consistent with the previous study [20]. In addition, we found that miR-129 was aberrantly downregulated in ESCC cells, and forced expression of miR-129 resulted in a marked repression on ESCC cell viability and invasion capacity. Similarly, previous documents revealed that miR-129 was frequently downregulated and functioned as a tumor suppressor in various tumors, such as gastric [35], colorectal [36], and lung cancer [37].

Several studies have stated that in the lncRNA-miRNA-mRNA regulatory network, lncRNAs serve as ceRNAs or miRNA sponges to interact with miRNAs at a posttranscriptional level, thereby reducing miRNA-mediated repression of their target mRNAs [38]. The balance of the lncRNA-miRNA-mRNA regulatory network is essential for many biological processes, and any disturbance of the ceRNA network may lead to different diseases including cancers [39]. Till now, NETA1 has been clarified to be linked with many cancers by acting as a ceRNA. For instance, NETA1 functioned as a ceRNA for miR-377-3p, antagonized its function, and led to the derepression of its endogenous target E2F3, which was a core oncogene in promoting non-small-cell lung carcinoma progression [40]. NEAT1 played an oncogenic role in the tumorigenesis of laryngeal squamous cell cancer by regulating the miR-107/CDK6 pathway [41]. Likewise, NEAT1 served as a molecular sponge for miR-449b-5p and led to the upregulation of its target c-Met, thus promoting glioma pathogenesis [19]. Therefore, we supposed that NEAT1 was involved in this network and acted as a miRNA sponge in ESCC. In the present study, NEAT1 actually contained a motif with sequence complementary to miR-129 and directly interacted with miR-129 in an Ago2-dependent manner, as demonstrated by bioinformatic analysis, luciferase reporter assay, and RIP. Also, NEAT1 was shown to suppress miR-129 expression in ESCC cells. These results demonstrated that NEAT1 functioned as an endogenous sponge to downregulate miR-129 by competitively binding to miR-129.

CTBP2, as a transcriptional corepressor, has been demonstrated to play an oncogenic role in tumorigenesis and progression in several tumors. For instance, CTBP2 was demonstrated to facilitate the development of ESCC [27] and breast cancer [42] through negatively regulating p16 (INK4A), a tumor suppressor gene product. Additionally, CTBP2 was found to be overexpressed in gastric cancer (GC) and correlated with poor prognosis and accelerate GC tumorigenesis and metastasis [25]. Furthermore, CTBP2 knockdown by lentiviral-mediated RNA interference resulted in inhibited cell growth, proliferation, migration, invasion, and cell cycle progression in neuroblastoma [43]. In the present study, to investigate whether NEAT1-induced miR-129 inhibition led to the derepression of its target mRNA, we focused on the miR-129 target gene CTBP2, which was found to be upregulated in ESCC cells in the current study. Bioinformatic analysis and luciferase reporter assay demonstrated that CTBP2 was a potential target of miR-129 and NEAT1 overexpression abolished miR-129-induced repression on luciferase activity of the CTBP2-WT reporter vector. Moreover, NEAT1 knockdown prominently decreased CTBP2 expression while anti-miR-129 abated this suppressive role, suggesting that NEAT1 could regulate the derepression of CTBP2 by inhibiting miR-129 expression. More importantly, CTBP2 restoration dramatically overturned cell viability and invasion suppression mediated by NEAT1 knockdown or miR-129 overexpression, indicating that the NEAT1-miR-129-CTBP2 regulatory network was involved in ESCC tumorigenesis.

## 5. Conclusions

Taken together, our study demonstrated that NEAT1 knockdown or miR-129 overexpression suppressed ESCC cell viability and invasion. Mechanistic analysis further uncovered the NEAT1-miR-129-CTBP2 regulatory axis involved in ESCC cell progression, providing new evidence that lncRNAs acted as ceRNAs to regulate the expressions and activities of miRNAs, thereby leading to the derepression of miRNA target mRNA. This study also suggested that targeting the NEAT1-miR-129-CTBP2 axis may be an effective therapeutic approach for ESCC.
